# Effect of Low-Dose, Long-Term Roxithromycin on Airway Inflammation and Remodeling of Stable Noncystic Fibrosis Bronchiectasis

**DOI:** 10.1155/2014/708608

**Published:** 2014-11-04

**Authors:** Jifeng Liu, Xiaoning Zhong, Zhiyi He, Lianghong Wei, Xiaozhen Zheng, Jianquan Zhang, Jing Bai, Wei Zhong, Dengjun Zhong

**Affiliations:** ^1^Department of Respiratory Disease, First Affiliated Hospital of Guangxi Medical University, No. 6 Shuangyong Road, Nanning 530021, China; ^2^Department of Respiratory Disease, Tenth Affiliated Hospital of Guangxi Medical University, Qinzhou 535000, China; ^3^Department of Radiology, Tenth Affiliated Hospital of Guangxi Medical University, Qinzhou 535000, China

## Abstract

*Background*. Noncystic fibrosis bronchiectasis (NCFB) is characterized by airway expansion and recurrent acute exacerbations. Macrolide has been shown to exhibit anti-inflammatory effects in some chronic airway diseases. *Objective*. To assess the efficacy of roxithromycin on airway inflammation and remodeling in patients with NCFB under steady state. *Methods*. The study involved an open-label design in 52 eligible Chinese patients with NCFB, who were assigned to control (receiving no treatment) and roxithromycin (receiving 150 mg/day for 6 months) groups. At baseline and 6 months, the inflammatory markers such as interleukin- (IL-)8, neutrophil elastase (NE), matrix metalloproteinase- (MMP)9, hyaluronidase (HA), and type IV collagen in sputum were measured, along with the detection of dilated bronchus by throat computed tomography scan, and assessed the exacerbation. *Results*. Forty-three patients completed the study. The neutrophil in the sputum was decreased in roxithromycin group compared with control (*P* < 0.05). IL-8, NE, MMP-9, HA, and type IV collagen in sputum were also decreased in roxithromycin group compared with the control group (all *P* < 0.01). Airway thickness of dilated bronchus and exacerbation were reduced in roxithromycin group compared with the control (all *P* < 0.05). *Conclusions*. Roxithromycin can reduce airway inflammation and airway thickness of dilated bronchus in patients with NCFB.

## 1. Introduction

Bronchiectasis is defined pathologically as a permanent dilatation of bronchi. Noncystic fibrosis bronchiectasis (NCFB) is an airway disease characterized by chronic inflammation, destruction of affected bronchi, and thickening of bronchial wall [1–3]. A combination of a defect in host defense and bacterial infection leads to microbial colonization of airway, resulting in chronic inflammation and lung damage [4–6].

Airway inflammation of patients with NCFB still exists at steady-state condition. In particular, matrix metalloproteinases- (MMP-) 9 and interleukin- (IL-) 8 which recruits neutrophil could lead to bronchial wall damage [3]. Over the past decade, there have been increasing interests in the nonantibiotic effects of macrolide antibiotics and their role in modulating disease activity in bronchiectasis. Macrolides could significantly reduce sputum volume, IL-8 levels in bronchoalveolar lavage fluid, total bronchoalveolar lavage cell counts, neutrophil ratios, and daily sputum production. Furthermore, macrolides could inhibit expression and activation of MMP-9 [7, 8]. In addition to the effect on sputum production and inhibition of cytokine release, macrolides appear to affect chronic* Pseudomonas* infection by suppressing its quorum sensing, thereby reducing the release of virulence factors [9, 10].

Our previous study showed that low-dose, long-term roxithromycin combined with ambroxol hydrochloride improved thorax CT scores in patients with bronchiectasis in stable condition [11]. Hence, it was hypothesized that roxithromycin could inhibit the inflammation and impact the dilated bronchial wall thickness of NCFB at its stable condition; meanwhile the St. George's Respiratory Questionnaire (SGRQ) scores and exacerbation could be reduced. The study involved a parallel control design of roxithromycin to assess its effects on inflammation media in induced sputum, dilated bronchial wall thickness, SGRQ scores, and exacerbation of patients with NCFB in stable condition.

## 2. Materials and Methods

### 2.1. Patients

Sixty-six Chinese patients aged between 18 and 65 years, who were hospitalized at the Tenth Affiliated Hospital of Guangxi Medical University directed by First Affiliated Hospital of Guangxi Medical University, Qinzhou, China, from May 2009 to July 2011, were enrolled in this study. The diagnosis of all patients was confirmed as bronchiectasis meeting the criteria of which O'Donnell et al. defined [1, 12]. The criteria are as follows: (a) standard chest radiograph compatible with bronchiectasis, for instance, fusiform infiltrates of mucoid impaction, “signet ring,” or “tram tracks,” (b) chest CT showing ectasia of peripheral bronchi, fluid-filled airways, or thickening of the mucosa, (c) patient with daily chronic sputum production or hemoptysis. The patients with exacerbation of bronchiectasis had been excluded according to a protocol-defined exacerbation (PDE) which O'Donnell et al. set down [12]. The PDE was prospectively defined as abnormalities in four of the following nine symptoms, signs, or laboratory findings: (1) change in sputum production (consistency, color, volume, or hemoptysis); (2) increased dyspnea (chest congestion or shortness of breath); (3) increased cough; (4) fever (≥38°C); (5) increased wheezing; (6) decreased exercise tolerance, malaise, fatigue, or lethargy; (7) forced expiratory volume in 1 second or forced vital capacity decreasing 10% from a previously recorded value; (8) radiographic changes indicative of a new pulmonary process; or (9) changes in chest sounds. Patients with cystic fibrosis (CF), who had documented clinical, radiologic, genotypic features and abnormal sweat test results (sweat sodium and chloride >60 mmol/L) [13], had been excluded. Patients who were allergic to macrolides and patients with impaired hepatic disease or diabetes mellitus had also been excluded.

### 2.2. Ethical Considerations

Study protocol and informed consent form were reviewed and approved by the hospital's Ethics Committee. Informed consent was obtained from all the patients before enrollment.

### 2.3. Study Design

All patients had a one-month run-in period free of exacerbation symptoms before baseline sampling. Eligible participants were randomly assigned to control and roxithromycin groups. Patients in the treatment group received oral open-label study of roxithromycin at 150 mg/day (dispersible tablets; Jiangsu Hengrui Medicine Co., Ltd., China) for 6 months, while the patients received no drug in control group. During the study period, patients were instructed to contact the study coordinator or investigator if they were having an allergic sign associated with roxithromycin, exacerbation of their underlying lung disease, and other side reactions (including headache, nausea, vomiting, and diarrhea) which the patients could not bear. Such patients were excluded from the study. Sputum was induced and examined at the beginning and after 6 months of the study. Thoracic HRCT scans of all the patients were performed at the beginning and after 6 months of the study. Inflammatory markers including IL-8, neutrophil elastase (NE), MMP-9, tissue inhibitor of metalloproteinases-1 (TIMP-1), hyaluronidase (HA), and type IV collagen concentration in induced sputum were recorded at baseline and after 6 months. Spirometry, quality of life, exacerbations, total sputum cells count, and differential cells count in induced sputum were recorded at baseline and after 6 months. Additionally, HRCT evaluation of each patient was detected at baseline and after 6 months. Treatment adherence was encouraged by telephone calls from the study coordinator and pill counts measurement.

### 2.4. Analysis of Sputum Samples

Sputum induction was performed per previously published methods [5, 14]. Subjects inhaled nebulized hypertonic saline at concentrations of 3, 4, and 5%, respectively. The induced sputum had been collected in culture dishes. The sample of induced sputum was qualified for the following criteria: leukocytes of >25 and squamous epithelial cell of <l0 in sputum counted by direct smear per low-power field [14]. Sputum was processed within 2 hours from collection. Sputum plugs processed with 4 × weight/volume of 0.1% dithiothreitol, to which 4 × weight/volume of phosphate-buffered saline was added. Samples were vortexed and incubated in a shaking water bath at 37°C for 15 min. The resulting suspensions were filtered through nylon gauze (48 *μ*m). Filtrates were centrifuged at 790 rpm/min for 10 min to remove the cells for counting the total cell counts and differential cells counts by the smear with Wright's staining. Supernatants were stored at −80°C until further analysis [15]. Sputum bacterial culture was performed at baseline and after 6 months of treatment. All samples of sputum were processed for bacterial culture within 4 h of expectoration. Viable bacterial numbers were expressed as the number of colony-forming units (cfu) per millilitre of original sputum [16].

### 2.5. Laboratory Assays

Concentrations of IL-8 and MMP-9 in the supernatant of induced sputum were assessed by enzyme-linked immunosorbent assay (ELISA) kits per manufacturer's manual (Human IL-8 ELISA Kit and Human MMP9 ELISA Kit, Shanghai ExCell Biology, Inc., China). Concentrations of NE and TIMP-1 in the supernatant of induced sputum were assessed by enzyme-linked immunosorbent assay (ELISA) kits per manufacturer's manual (Human NE ELISA Kit and Human TIMP-1 ELISA Kit, Wuhan Boster Biology, Inc., China). The sensitivities of assays as quoted by the manufacturer were 15 pg/mL and 10 pg/mL, respectively. HA and type IV collagen concentrations were measured in sputum samples using a radioimmunoassay (RIA) per manufacturer's manual (RIA Kits, Beijing North Institute of Biological Technology, China).

### 2.6. HRCT Evaluation

The thoracic HRCT scans were performed on a SOMATOM EMOTION 16 CT (Siemens Somatom, Germany) scanner with a collimation of 1–1.5 mm, and the images were reconstructed using a high spatial frequency algorithm and 512 × 512 matrices. The scan parameters included time of 1 second, voltage of 130 kV, and current of 200 mAs. The images were analyzed on a window width of 1500 Hounsfield units (HU) and window level of −600 HU [17]. Data were collected at the five levels on full inspiration with additional scans on expiration for assessment of air trapping and mosaic perfusion. The images were viewed using Image-Pro Plus 6.0 (Media Cybernetics, Inc., USA) at a magnification of ×3. Evaluated CT scores of each patient were defined per the protocol as described by Ooi et al. [17]. The protocol was defined to evaluate CT scores of each patient on the following assessments: each lobe (i.e., the lingula segment was considered as a separate lobe) of the lungs was separately evaluated for the extent of bronchiectasis and bronchial wall thickening. The extent of bronchiectasis was quantified by first assigning a score to each of the six lobes per the percentage (i.e., grade) of lobar involvement, which was derived with the following scale: grade 0, none; grade 1, mild (0–25%); grade 2, moderate (25%–50%); and grade 3, severe (>50% involvement of each lobe). All individual lobar scores were summed to calculate the overall score for the extent of bronchiectasis. The thickness of the bronchial wall relative to the external diameter of dilated bronchi (EDB) perpendicular to the transverse plane was evaluated in each lobe. This score was determined with the following scale: grade 0, normal thickness; grade 1, thickness >20% and <50% EDB; grade 2, thickness >50% EDB; and grade 3, complete obliteration of the bronchial lumen. If there was a range of bronchial wall thickening noted in each lobe assessed, a mean score was calculated per lobe, whereby the number of scores assigned was the denominator for the sum of all scores calculated. The sum of individual lobar bronchial wall thickening scores was the overall score for each patient. In each lobe, the presence of small-airway abnormalities and mosaic pattern was, respectively, assessed as grade 1, when these findings were considered present, and grade 0, when they were considered absent. Individual lobar scores were summed to calculate the total score for small-airway abnormalities and mosaic attenuation for each patient. A global bronchiectasis score including all four HRCT scores was also calculated for analysis. The evaluation of CT scores was conducted by two experienced thoracic radiologists, who worked independently in a blinded fashion.

**Figure 1:**
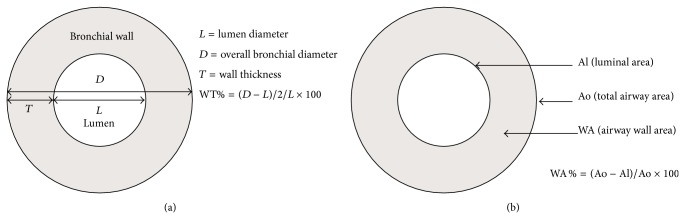
Evaluation of WT% and WA%.

**Figure 2:**
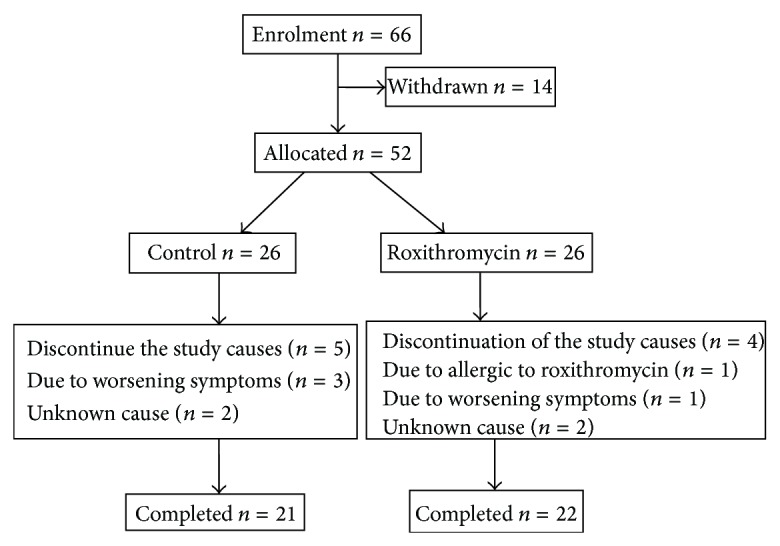
Subject disposition.

The measurements of overall (*D*1, *D*2, and *D*3) and internal (*L*1, *L*2, and *L*3) diameters of the abnormal dilated bronchi were made using electronic calipers from three different directions and cross the centre of the bronchi, with mean wall thickness (*T*) being derived from the measurement of [*T* = (*D*1 + *D*2 + *D*3 − *L*1 − *L*2 − *L*3)/2] [18] (Figure 1(a)). Total airway area (Ao) and luminal area (Al) were also measured (Figure 2). Five bronchi were selected randomly and were measured and calculated at each slice of the scans. Percentage wall thickness (WT%) was defined as [(*D* − *L*)/2/*D*] × 100, and percentage wall area (WA%) was defined as [(Ao − Al)/Ao] × 100. Both were calculated as shown in Figure 1(b). The measurements were conducted by two experienced thoracic radiologists who worked independently in a blinded fashion. A mean value was calculated for each patient for each observer from 5 bronchi measured, which were selected randomly at each slice of the scans [19].

### 2.7. Quality of Life

Health status was measured using the SGRQ at baseline and after 6 months [20].

### 2.8. Monitoring and Definition of Exacerbation

The patients with exacerbation of bronchiectasis had been defined according to a protocol-defined exacerbation (PDE) which O'Donnell et al. set down [12]. Information on exacerbations was collected during clinic visits, and any patient experiencing worsening respiratory symptoms was instructed to contact the investigator immediately and report to the study clinic as soon as possible. The clinic visits for exacerbation of all patients had been proceeded for 12-months.

### 2.9. Safety Evaluation

Physical examinations were performed at entry and trial completion of the study. Routine biochemistry and hematology tests were also assessed at study entry and after 6 months. The occurrence of adverse events including fever, headache, nausea, vomiting, diarrhea, and skin rashes was monitored and recorded at each study visit and compared between the groups.

### 2.10. Statistical Analysis

Statistical analyses were performed using SPSS 16.0 for Windows (SPSS Inc., USA). All data were expressed as mean ± standard deviation (SD) or median (M) with the range. Comparison of gender and present smokers between both groups was performed using x^2^ inspection, and comparison of CT scores and SGRQ scores between both groups was performed using Mann-Whitney test. For statistical analysis, longitudinal SGRQ scores of 6 months from baseline was performed using two related samples test analysis. Comparison of the baseline measurement data and 6-month measurement data between both groups was analyzed using two-sample *t* tests including age; body mass index; patient's disease history; smoking history; spirometry; cells counts in sputum; concentrations of IL-8, NE, MMP9, TIMP-1, MMP9/TIMP-1, HA, and type IV collagen in induced sputum; and WT% and WA% of CT evaluation. For statistical analysis, longitudinal measurement data of 6 months from baseline was performed using paired *t* test analysis. To assess the correlation between WT% and WA% of CT evaluation and concentrations of IL-8, NE, MMP9, HA, and type IV collagen in induced sputum at baseline using Pearson correlation for statistical analysis, respectively, *P* value <0.05 (two-tailed tests) was considered statistically significant.

## 3. Results

Fifty-two eligible patients were enrolled in the study. Of them, 26 were assigned to receive nothing in control group and 26 were assigned to receive roxithromycin in treatment group. A total of 43 patients completed the study (Figure 2).

### 3.1. Demographic and Baseline Characteristics

There were no significant differences between both study groups at baseline with respect to age, gender, body mass index (BMI), patient's disease history, smoking status, spirometry, and CT scores (*P* > 0.05) (Table 1).

### 3.2. Sputum Bacteriology

At baseline, eight patients with NCFB had bacterial growth in the sputum, of which four patients had more than one organism. The main two bacterial pathogens in the sputum were* Pseudomonas aeruginosa *(3 each in control and roxithromycin groups) and* Haemophilus influenzae *(2 in roxithromycin group). At the 6-month time point, 7 sputum samples from the patients had significant bacterial growth and 3 of these specimens had growth of more than one organism. The main three detected pathogens were* P. aeruginosa* (2 each in control and roxithromycin groups) and* mycoplasmata* (1 in control and 2 in roxithromycin group). There was no difference in the detection rate of three main microorganisms between the two groups at baseline or after 6 months of treatment. Interestingly, nontuberculosis mycobacteria (NTM) could not be detected in patients of both groups.

### 3.3. Sputum Cell Counts

Baseline total sputum counts and differential cell counts were similar between the two groups (Table 2). Treatment with roxithromycin significantly decreased the total number of cells in induced sputum from baseline to 6 months (*P* < 0.001). This reduction was also significantly greater compared with control (*P* = 0.006). Roxithromycin also produced a similar reduction in neutrophil cell counts in induced sputum from baseline to 6 months (*P* < 0.001, Table 2), and this decrease was significantly compared with that of control (*P* = 0.037, Table 2). In contrast, treatment with roxithromycin did not significantly decrease macrophages and lymphnocytes cell counts in induced sputum compared with those of control (*P* = 0.415, *P* = 0.181, resp.; Table 2).

### 3.4. Inflammatory Markers in Sputum

**Figure 3:**
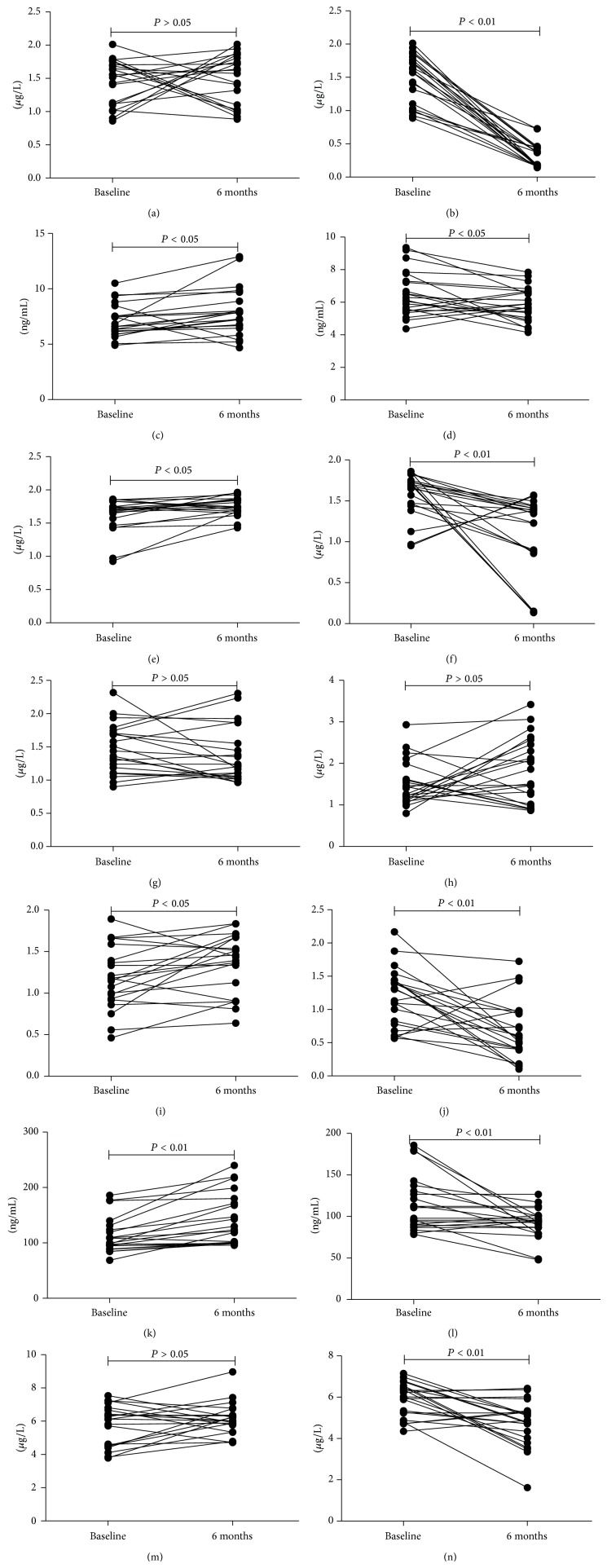
Variation of IL-8, NE, MMP-9, TIMP-1, MMP9/TIMP-1, HA, and type IV collagen in induced sputum from baseline to 6 months in both groups. (a) There was no statistical difference in IL-8 from baseline to 6 months in controls; (b) effect of roxithromycin on induced sputum IL-8 concentration (*μ*g/L) in patients with NCFB from baseline to 6 months of treatment (*P* < 0.001); (c) induced sputum NE concentration (ng/mL) in patients with NCFB increasing from baseline to 6 months in controls (*P* = 0.043); (d) effect of roxithromycin on induced sputum NE concentration (ng/mL) in patients with NCFB from baseline to 6 months of treatment (*P* = 0.029); (e) induced sputum MMP-9 concentration (*μ*g/L) in patients with NCFB increasing from baseline to 6 months in controls (*P* = 0.011); (f) effect of roxithromycin on induced sputum MMP-9 concentration (*μ*g/L) in patients with NCFB from baseline to 6 months of treatment (*P* = 0.002); (g) there was no statistical difference in TIMP-1 from baseline to 6 months in controls; (h) there was no statistical difference in TIMP-1 from baseline to 6 months in roxithromycin; (i) there was no statistical difference in MMP9/TIMP-1 from baseline to 6 months in controls; (j) effect of roxithromycin on induced sputum MMP9/TIMP-1 in patients with NCFB from baseline to 6 months of treatment (*P* = 0.005); (k) induced sputum HA concentration (ng/mL) in patients with NCFB increased from baseline to 6 months in controls (*P* = 0.011); (l) effect of roxithromycin on induced sputum hyaluronidase concentration (ng/mL) in patients with NCFB from baseline to 6 months of treatment (*P* = 0.005); (m) there was no statistical difference in sputum type IV collagen concentration from baseline to 6 months in controls; (n) effect of roxithromycin on induced sputum type IV collagen concentration (*μ*g/L) in patients with NCFB from baseline to 6 months of treatment (*P* < 0.001).

**Figure 4:**
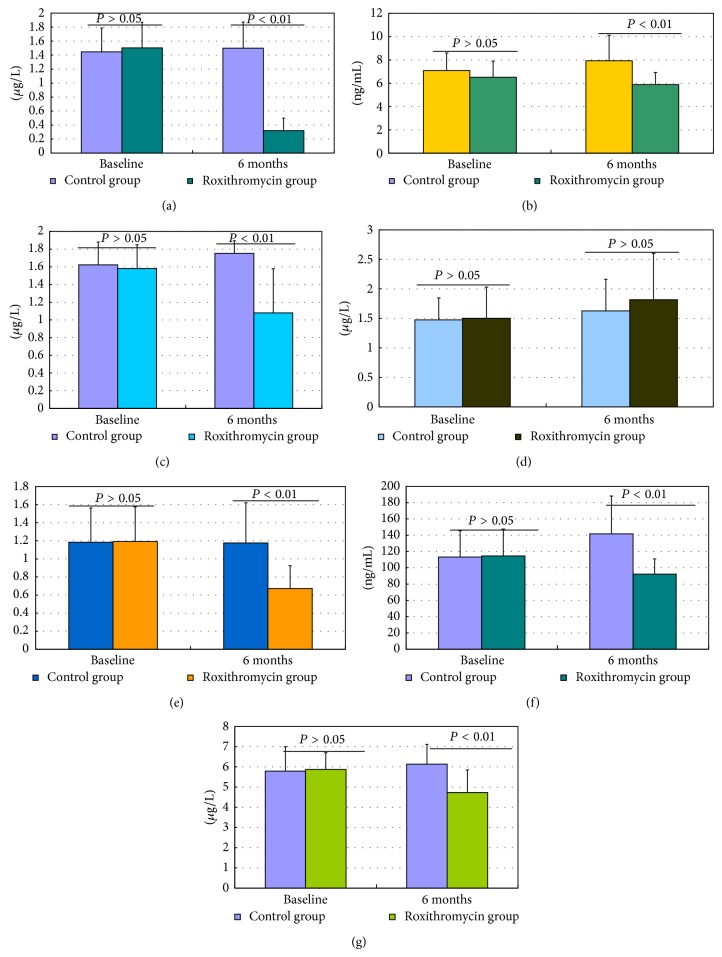
Effect of roxithromycin on inflammatory marker in sputum of patients after 6 months of treatment. (a) Effect of roxithromycin on induced sputum IL-8 concentration (*μ*g/L) in patients with NCFB after 6 months of treatment (*P* < 0.001); (b) effect of roxithromycin on induced sputum NE concentration (ng/mL) in patients with NCFB after 6 months of treatment (*P* = 0.001); (c) effect of roxithromycin on induced sputum MMP-9 concentration (*μ*g/L) in patients with NCFB after 6 months of treatment (*P* < 0.001); (d) there were no significant changes of activity scores and impact scores in either group (*P* > 0.05); (e) effect of roxithromycin on induced sputum MMP9/TIMP-1 in patients with NCFB after 6 months of treatment (*P* < 0.001); (f) effect of roxithromycin on induced sputum HA concentration (ng/mL) in patients with NCFB after 6 months of treatment (*P* < 0.001); (g) effect of roxithromycin on type IV collagen concentration (*μ*g/L) in patients with NCFB after 6 months of treatment (*P* < 0.001).

The concentrations of NE, MMP9, MMP9/TIMP-1, and hyaluronidase in the supernatant of induced sputum significantly increased from baseline to 6 months in control group (*P* = 0.043, *P* = 0.011, *P* = 0.019, and *P* < 0.001, resp.; Figures 3(c) and 3(e)). There were significantly decreased concentrations of IL-8, NE, MMP9, MMP9/TIMP-1, hyaluronidase, and collagen type IV in the supernatant of induced sputum from baseline to 6 months in roxithromycin group (*P* < 0.001, *P* = 0.029, *P* = 0.002, *P* = 0.005, *P* = 0.005, and *P* < 0.001, resp.; Figures 3(b), 3(d), 3(f), and 3(h)). There was no statistical difference in IL-8, NE, MMP9, TIMP-1, MMP9/TIMP-1, HA, and type IV collagen between the two groups at baseline (*P* > 0.05) (Figures 4(a), 4(b), 4(c), and 4(d)). However, the concentrations of IL-8, NE, MMP9, MMP9/TIMP-1, HA, and type IV collagen in induced sputum were also significantly reduced in roxithromycin group as compared with control at the end of 6 months (all *P* < 0.01, resp.; Figures 4(a), 4(b), 4(c), and 4(d)). There was no significant change of TIMP-1 in either group (*P* > 0.05).

### 3.5. Effect of Roxithromycin on WT% and WA%

**Figure 5:**
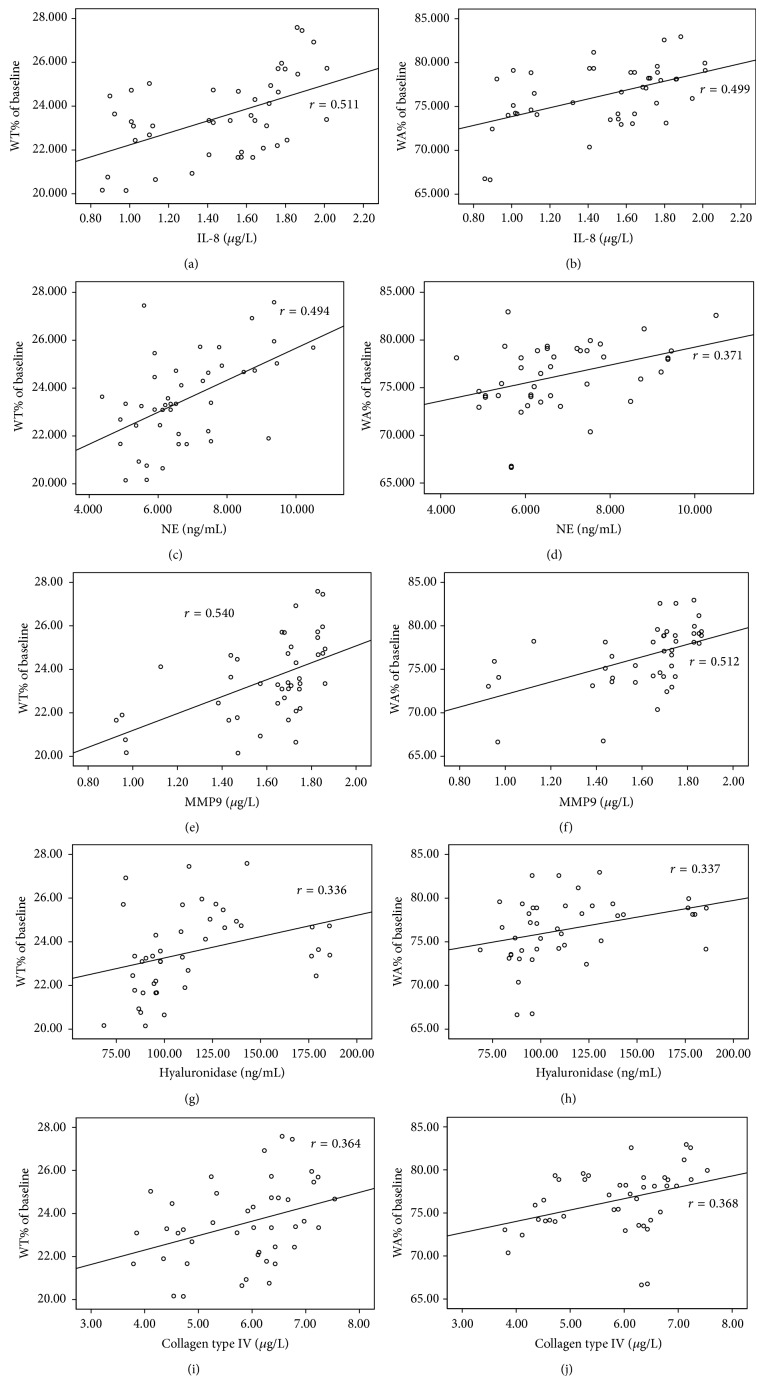
Correlation of WT% and WA% with IL-8, MMP9, HA, and type IV collagen. (a) Correlation between the concentration of IL-8 in induced sputum and WT%; (b) correlation between the concentration of IL-8 in induced sputum and WA%; (c) correlation between the concentration of NE in induced sputum and WT%; (d) correlation between the concentration of NE in induced sputum and WA%; (e) correlation between the concentration of MMP9 in induced sputum and WT%; (f) correlation between the concentration of MMP9 in induced sputum and WA%; (g) correlation between the concentration of HA in induced sputum and WT%; (h) correlation between the concentration of HA in induced sputum and WA%; (i) correlation between the concentration of type IV collagen in induced sputum and WT%; (j) correlation between the concentration of type IV collagen in induced sputum and WA%.

**Figure 6:**
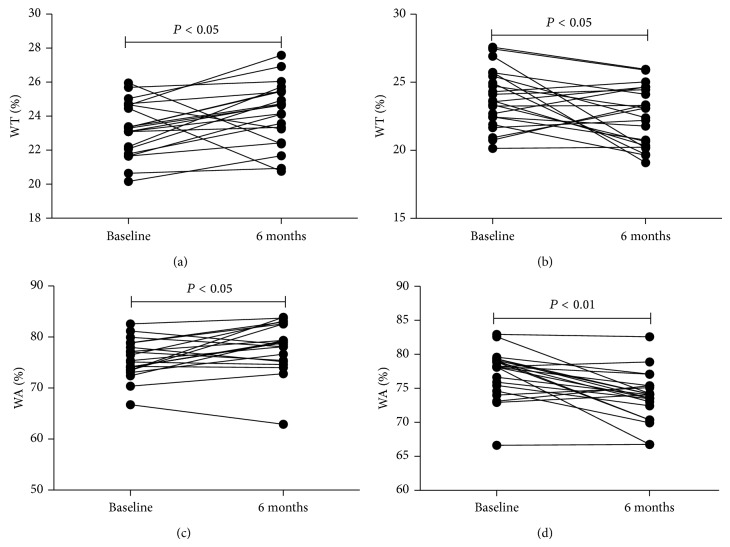
(a) WT% in patients with NCFB increased from baseline to 6 months in controls (*P* = 0.024); (b) effect of roxithromycin on WT% in patients with NCFB from baseline to 6 months of treatment (*P* = 0.018); (c) WA% in patients with NCFB increased from baseline to 6 months in controls (*P* = 0.014); and (d) effect of roxithromycin on WA% in patients with NCFB from baseline to 6 months of treatment (*P* < 0.001).

**Figure 7:**
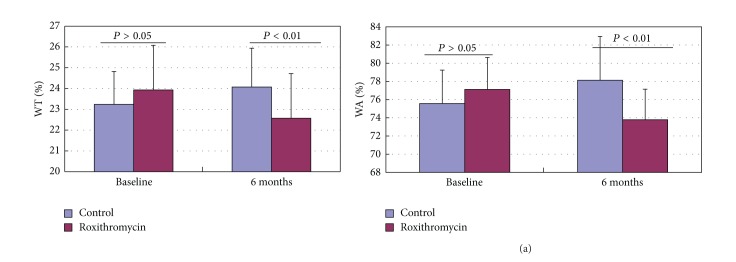
Effect of roxithromycin on WT% and WA% in patients with NCFB at baseline and after 6 months of treatment (*P* = 0.018 and *P* = 0.001, resp.; roxithromycin versus control after 6 months of treatment).

WT% was positively correlated with IL-8 (*r* = 0.542, *P* < 0.001), NE (*r* = 0.494, *P* = 0.001), MMP-9 (*r* = 0.540, *P* < 0.001), HA (*r* = 0.336, *P* = 0.028), and type IV collagen (*r* = 0.364, *P* = 0.016) in induced sputum of all patients in both groups at baseline (Figures 5(a), 5(c), 5(e), 5(g), and 5(i)). WA% was also positively correlated with IL-8 (*r* = 0.499, *P* = 0.001), NE (*r* = 0.371, *P* = 0.014), MMP-9 (*r* = 0.512, *P* < 0.001), HA (*r* = 0.337, *P* = 0.027), and type IV collagen (*r* = 0.368, *P* = 0.015) in induced sputum of all patients in both groups at baseline (Figures 5(b), 5(d), 5(f), 5(h), and 5(j)). There was no statistical difference in WT% and WA% between the two groups on baseline (*P* > 0.05). WT% and WA% had increased significantly from baseline to 6 months in control (*P* = 0.024 and *P* = 0.014, resp.; Figures 6(a) and 6(c)). WT% and WA% had decreased significantly from baseline to 6 months in roxithromycin (*P* = 0.018 and *P* < 0.001, resp.; Figures 6(b) and 6(d)); and, furthermore, WT% and WA% had decreased significantly as roxithromycin group compared with control group on 6 months (*P* = 0.018 and *P* = 0.001, resp.; Figures 6 and 7(a)).

### 3.6. Health-Related Quality of Life

SGRQ scores were not significantly different at baseline between the control and roxithromycin groups. In the roxithromycin group, total scores in the SGRQ had significantly improved after 6 months (*P* = 0.02) compared with baseline (Table 3). Similarly, there was also a significant reduction in symptom scores after 6 months compared with baseline (*P* = 0.045 and *P* = 0.044, resp.). Furthermore, the total scores and symptom scores had significantly improved in roxithromycin group after 6 months as compared with control group. However, there were no significant changes of activity scores and impact scores in either group (Table 3).

### 3.7. Exacerbations

There were a total of 27 acute exacerbations after the 6-month treatment time, of which 16 occurred in the control group and 11 in the roxithromycin group. The proportion of patients with at least one exacerbation was 76.2% in the control group and 50% in the roxithromycin group. The median time to the first exacerbation was 113 days in the control group and 264 days in the roxithromycin group. Kaplan-Meier survival analysis showed that roxithromycin significantly delayed the time to the first NCFB exacerbation compared with control (*P* = 0.022; log-rank test; Figure 9).

### 3.8. Safety Evaluation

Two patients in both groups were lost to follow-up due to unknown reason. Five patients experienced nausea at first week in roxithromycin group, but all of them could tolerate the treatment for 6 months. Three patients in the control group and one patient in the roxithromycin group had an exacerbation. One patient in roxithromycin presented skin rashes which was an allergic reaction to roxithromycin. One patient, who received roxithromycin therapy for 6 months, had experienced mycoplasmata in urine, which was resistant to roxithromycin because of urinary tract infection. No other adverse reactions had been found in patients during 1-year follow-up after receiving roxithromycin therapy for 6 months [21].

## 4. Discussion

The present study aimed to investigate the efficacy of roxithromycin in suppressing airway inflammation and chronic remodeling of dilated bronchial wall in patients with NCFB under steady state and furthermore investigate the impact of treatment with roxithromycin on SGRQ scores and the time to the first NCFB exacerbation. The study results provided clinically relevant information on the treatment of NCFB using macrolides.

Induced sputum is a technique that has been used to investigate cellular and cytokine changes in response to oral and inhaled steroids. The present study showed a reduction in the total cell number in sputum and neutrophils, after roxithromycin treatment. Furthermore, the treatment with roxithromycin also significantly reduced IL-8, NE, MMP-9, MMP9/TIMP-1, HA, and type IV collagen concentrations in sputum; and it decreased the thickness of dilated bronchial wall in patients with steady-state NCFB, along with its reduced SGRQ scores and acute exacerbation. Meanwhile, WT% and WA% were positively correlated with IL-8, NE, MMP-9, MMP9/TIMP-1, HA, and type IV collagen concentrations in sputum. These showed that IL-8, NE, MMP9, HA, and type IV collagen concentrations in sputum associated with remodeling of dilated bronchial wall in steady-state NCFB. It may suggest that inflammation can damage bronchial wall, which leads to infiltration of inflammatory cells in bronchia and disposition of type IV collagen in bronchial wall and promotes remodeling of affected bronchia. This parallel open-label, control study in patients with NCFB showed beneficial effects of roxithromycin on airway inflammation, remodeling of NCFB, and the time to the first NCFB exacerbation of NCFB in stable condition.

Early in the decade years ago,it had been demonstrated that roxithromycin could decrease the degree of airway responsiveness in patients with bronchiectasis [22]. Recent several clinical studies documented that azithromycin and low-dose erythromycin decreased exacerbations and infections in patients with NCFB [23–26]. This study also showed that roxithromycin delayed the time to the first NCFB exacerbation. Several clinical studies demonstrated that macrolide has beneficial effects on NCFB. However, they did not show whether macrolide could affect inflammation and structure of NCFB. Other recent clinical trials and our previous clinical trial showed that macrolide could improve lung function and CT score [11, 27]. Hence, these results have suggested that macrolide possesses beneficial treatment efficacy in NCFB, and it can reduce colony of microorganisms and inhibit inflammation. However, these clinical studies did not illustrate the mechanism of macrolide action, which can improve lung function and CT score.

The study results showed that roxithromycin treatment reduced airway inflammation, SGRQ scores, acute exacerbation, and remodeling of dilated bronchial wall in patients with NCFB, as shown by a decreased number of neutrophils and concentration of IL-8, NE, MMP-9, MMP9/TIMP-1, and HA in induced sputum. On the other hand, the study showed that the concentrations of NE, MMP-9, and HA in induced sputum significantly increased from baseline to 6 months without roxithromycin treatment. A study showed that NCFB inflammatory process could develop at stable condition. The activation of NE, MMP-9, and HA might lead to gelatinolytic and type IV collagenolytic processes, which were ascribed by a chronic active role in the disease process and were associated with a chronic bronchial wall damage, leading to increase in type IV collagen concentration [3, 6, 28, 29]. The increasing type IV collagen may result in deposition on bronchial wall and lead to thickening of the affected bronchial wall and obstruction of airway [2, 28–30]. This may also induce a beneficial effect by reducing proteolytic damage in the airways. It has been documented that macrolides significantly reduced sputum volume, IL-8 levels in bronchoalveolar lavage fluid, total bronchoalveolar lavage cell counts, neutrophil ratios, and daily sputum production. Furthermore, macrolides could inhibit expression and activation of NE and MMP-9 [7, 8, 31, 32]. Our previous study showed that erythromycin, belonging to macrolide antibiotics, had reduced HA and type IV collagen in chronic bronchitis and emphysema rat model, which induced lipopolysaccharide and cigarette smoking [33]. As the present study showed that affected airway thickness positively correlated with NE, MMP-9, HA, and type IV collagen and roxithromycin could decrease the affected airway thickness and reduce NE, MMP-9, HA, and along with type IV collagen, recent researches suggested that macrolide could improve CT score in NCFB [11, 27]. These suggested that low-dose, long-term treatment with roxithromycin may suppress airway inflammation and then reduce chronic damage activity on bronchus in patients with NCFB at stable condition, and it may also reduce NCFB exacerbation by suppressing airway inflammation and chronic damage activity.

The present study has some limitations. First, this is a single center trial with small sample size. Hence, a further multicenter trial with large sample size is warranted. Second, the current study has an open-label parallel control design. Third, adverse reactions of macrolide need further monitoring and detection on multicenter trials with large sample size. Most importantly, the macrolide-resistant bacteria should be further studied, because macrolide is the most important driver for the emergence of macrolide resistance in patients for long-term application [21].

**Figure 8:**
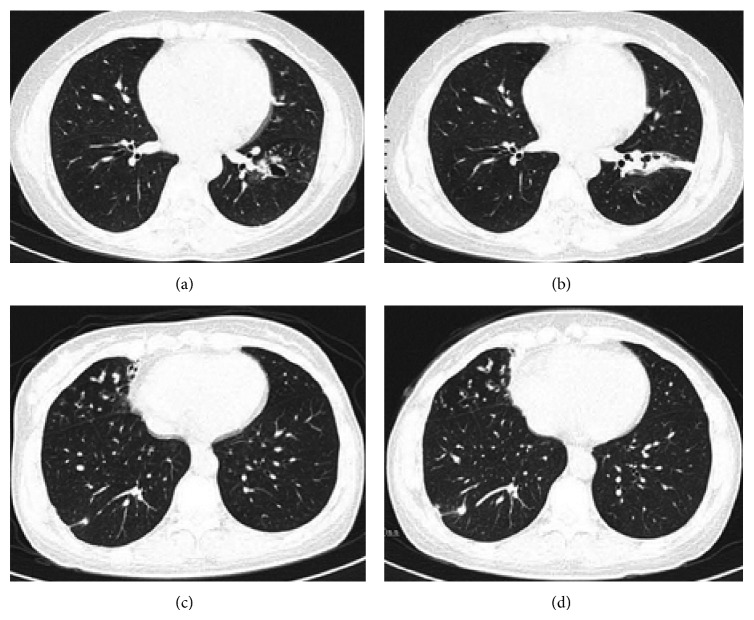
Variation of airway thickness of dilated bronchus in NCFB on CT scan. ((a), (b)) Airway thickness of the affected bronchi increased in control group and ((c), (d)) airway thickness of the affected bronchi decreased in roxithromycin group.

**Figure 9:**
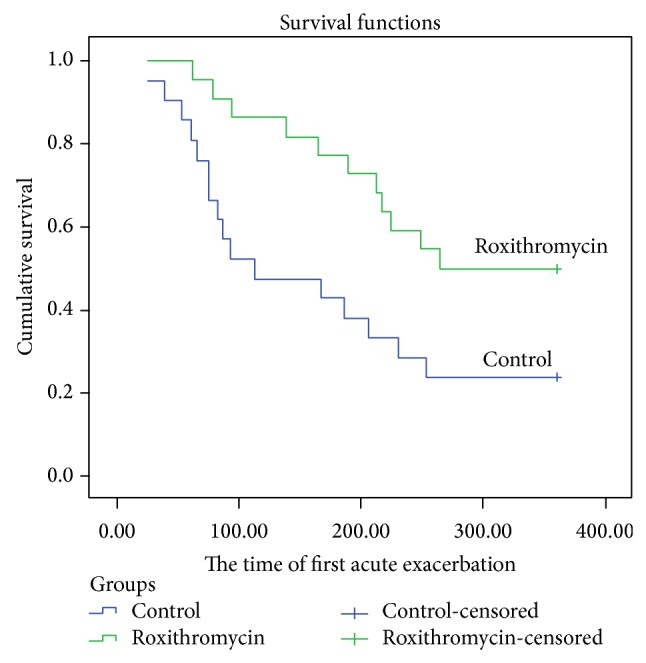
Kaplan-Meier curves showing the proportion of patients without an exacerbation (cumulative survival analysis) compared to time to the first exacerbation for the placebo and roxithromycin groups (*P* = 0.022).

To summarize, the treatment with roxithromycin can decrease airway inflammation and reduce airway thickness of dilated bronchus, which are positively associated with chronic airway inflammation in steady-state NCFB (Figure 8). However, further multicenter, randomized, placebo-controlled studies are required to confirm the effect of macrolide on steady-state NCFB.

## Figures and Tables

**Table 1:** Demographic and baseline characteristics.

Parameters	Control group	Roxithromycin group	*P* value
Sex (M : F)	21 (12 : 9)	22 (11 : 11)	0.157
Age (years)	49.2 ± 9.1	47.1 ± 7.9	0.423
Body mass index	23.38 ± 3.95	23.87 ± 3.62	0.674
Patient's disease history (years)	7.3 ± 2.9	8.7 ± 3.2	0.141
Smoking status			
Present smokers	3	5	0.4763
Smoking history (pack-years)	4.3 ± 0.97	4.7 ± 1.13	0.2210
Spirometry			
FEV_1_ (L)	1.63 ± 0.42	1.59 ± 0.37	0.7417
Percentage of predicted FEV_1_	67.4 ± 12.1	66.8 ± 10.9	0.8651
FVC (L)	2.34 ± 0.63	2.27 ± 0.59	0.7087
FEV_1_/FVC	69.6 ± 13.1	70.0 ± 13.5	0.9220
High-resolution CT parameter			
Small-airway abnormalities	3.09 ± 1.34	2.92 ± 0.97	0.268
Bronchial wall thickening	4.68 ± 2.01	4.79 ± 2.25	0.862
Extent of bronchiectasis	1.76 ± 0.43	1.76 ± 0.45	0.967
Global CT score	9.54 ± 3.56	9.47 ± 3.32	0.788

CT: computed tomography.

**Table 2:** Effect of roxithromycin on the inflammatory cell (10^6^/mL) in induced sputum inflammatory markers in sputum.

Parameters	Group	Baseline	6 months	*P* value 6 months versus baseline	*P* value versus control at baseline	*P* value versus control at 6 months
Total cells	Control	6.156 ± 0.494	6.495 ± 0.444	0.069		
	Roxithromycin	6.349 ± 0.502	5.773 ± 0.403^a^	0.001	0.211	<0.001

Neutrophils	Control	4.384 ± 0.436	4.687 ± 0.372	0.055		
	Roxithromycin	4.586 ± 0.420	4.051 ± 0.400^a^	<0.001	0.129	<0.001

Macrophages	Control	0.893 ± 0.095	0.931 ± 0.072	0.079		
	Roxithromycin	0.901 ± 0.088	0.898 ± 0.085	0.878	0.762	0.170

Lymphocytes	Control	0.879 ± 0.094	0.877 ± 0.098	0.915		
	Roxithromycin	0.861 ± 0.125	0.824 ± 0.129	0.297	0.606	0.142

^a^ *P* < 0.01 (*P* = 0.001 and *P* < 0.001) compared with 6 months of controls.

**Table 3:** Effects of roxithromycin on SGRQ scores in NCFB.

Parameters	Group	Baseline	6 months	*P* value 6 months versus baseline	*P* value versus control at baseline	*P* value versus control at 6 months
Total score	Control	58.3 ± 15.4	55.4 ± 15.0	0.326		
	Roxithromycin	56.7 ± 14.8	42.7 ± 13.5^a^	0.013	0.367	0.021

Symptom	Control	66.3 ± 12.5	64.0 ± 11.7	0.145		
	Roxithromycin	67.5 ± 16.9	60.5 ± 10.9^a^	0.016	0.276	0.035

Activity	Control	56.1 ± 12.2	55.6 ± 10.7	0.293		
	Roxithromycin	57.3 ± 10.8	52.4 ± 9.3	0.072	0.473	0.237

Impact	Control	36.3 ± 5.7	36.5 ± 6.6	0.702		
	Roxithromycin	35.6 ± 5.2	33.4 ± 4.9	0.224	0.406	0.324

^a^ *P* < 0.05 compared with 6 months of controls.

## References

[1] O'Donnell A. E. (2008). Bronchiectasis. *Chest*.

[2] King P. T. (2009). The pathophysiology of bronchiectasis. *International Journal of Chronic Obstructive Pulmonary Disease*.

[3] Bergin D. A., Hurley K., Mehta A., Cox S., Ryan D., O'Neill S. J., Reeves E. P., McElvaney N. G. (2013). Airway inflammatory markers in individuals with cystic fibrosis and non-cystic fibrosis bronchiectasis. *Journal of Inflammation Research*.

[4] King P. (2011). Pathogenesis of bronchiectasis. *Paediatric Respiratory Reviews*.

[5] Baydarian M., Walter R. N. (2008). Bronchiectasis: introduction, etiology, and clinical features. *Disease-a-Month*.

[6] Fuschillo S., De Felice A., Balzano G. (2008). Mucosal inflammation in idiopathic bronchiectasis: cellular and molecular mechanisms. *European Respiratory Journal*.

[7] Friedlander A. L., Albert R. K. (2010). Chronic macrolide therapy in inflammatory airways diseases. *Chest*.

[8] Kanai K.-I., Asano K., Hisamitsu T., Suzaki H. (2004). Suppression of matrix metalloproteinase-9 production from neutrophils by a macrolide antibiotic, roxithromycin, in vitro. *Mediators of Inflammation*.

[9] Ilowite J., Spiegler P., Chawla S. (2008). Bronchiectasis: new findings in the pathogenesis and treatment of this disease. *Current Opinion in Infectious Diseases*.

[10] Hill A. T., Pasteur M., Cornford C., Welham S., Bilton D. (2011). Primary care summary of the British Thoracic Society Guideline on the management of non-cystic fibrosis bronchiectasis. *Primary Care Respiratory Journal*.

[11] Liu J.-F., Zhong X.-N., He Z.-Y., Zhong D.-J., Bai J., Zhang J.-Q., Zhong W. (2012). Impact of treatment with low dose roxithromycin on stable bronchiectasis. *Zhonghua Jie He He Hu Xi Za Zhi*.

[12] O'Donnell A. E., Barker A. F., Ilowite J. S., Fick R. B. (1998). Treatment of idiopathic bronchiectasis with aerosolized recombinant human DNase I. *Chest*.

[13] Judge E. P., Dodd J. D., Masterson J. B., Gallagher C. G. (2006). Pulmonary abnormalities on high-resolution CT demonstrated more rapid decline than FEV_1_ in adults with cystic fibrosis. *Chest*.

[14] Pizzichini E., Pizzichini M. M. M., Efthimiadis A., Evans S., Morris M. M., Squillace D., Gleich G. J., Dolovich J., Hargreave F. E. (1996). Indices of airway inflammation in induced sputum: reproducibility and validity of cell and fluid-phase measurements. *The American Journal of Respiratory and Critical Care Medicine*.

[15] Gray R. D., MacGregor G., Noble D., Imrie M., Dewar M., Boyd A. C., Innes J. A., Porteous D. J., Greening A. P. (2008). Sputum proteomics in inflammatory and suppurative respiratory diseases. *The American Journal of Respiratory and Critical Care Medicine*.

[16] Pye A., Stockley R. A., Hill S. L. (1995). Simple method for quantifying viable bacterial numbers in sputum. *Journal of Clinical Pathology*.

[17] Ooi G. C., Khong P. L., Chan-Yeung M., Ho J. C. M., Chan P. K. S., Lee J. C. K., Lam W. K., Tsang K. W. T. (2002). High-resolution CT quantification of bronchiectasis: clinical and functional correlation. *Radiology*.

[18] Little S. A., Sproule M. W., Cowan M. D., Macleod K. J., Robertson M., Love J. G., Chalmers G. W., McSharry C. P., Thomson N. C. (2002). High resolution computed tomographic assessment of airway wall thickness in chronic asthma: reproducibility and relationship with lung function and severity. *Thorax*.

[19] Kasahara K., Shiba K., Ozawa T., Okuda K., Adachi M. (2002). Correlation between the bronchial subepithelial layer and whole airway wall thickness in patients with asthma. *Thorax*.

[20] Jones P. W., Quirk F. H., Baveystock C. M., Littlejohns P. (1992). A self-complete measure of health status for chronic airflow limitation. The St. George's Respiratory Questionnaire. *The American Review of Respiratory Disease*.

[21] Malhotra-Kumar S., Lammens C., Coenen S., van Herck K., Goossens H. (2007). Effect of azithromycin and clarithromycin therapy on pharyngeal carriage of macrolide-resistant streptococci in healthy volunteers: a randomised, double-blind, placebo-controlled study. *The Lancet*.

[22] Koh Y. Y., Lee M. H., Sun Y. H., Sung K. W., Chae J. H. (1997). Effect of roxithromycin on airway responsiveness in children with bronchiectasis: a double-blind, placebo-controlled study. *European Respiratory Journal*.

[23] Serisier D. J., Martin M. L. (2011). Long-term, low-dose erythromycin in bronchiectasis subjects with frequent infective exacerbations. *Respiratory Medicine*.

[24] Wong C., Jayaram L., Karalus N., Eaton T., Tong C., Hockey H., Milne D., Fergusson W., Tuffery C., Sexton P., Storey L., Ashton T. (2012). Azithromycin for prevention of exacerbations in non-cystic fi brosis bronchiectasis (EMBRACE): a randomised, double-blind, placebo-controlled trial. *The Lancet*.

[25] Altenburg J., de Graaff C. S., Stienstra Y., Sloos J. H., Van Haren E. H. J., Koppers R. J. H., van Der Werf T. S., Boersma W. G. (2013). Effect of azithromycin maintenance treatment on infectious exacerbations among patients with non-cystic fibrosis bronchiectasis: the BAT randomized controlled trial. *The Journal of the American Medical Association*.

[26] Serisier D. J., Martin M. L., McGuckin M. A., Lourie R., Chen A. C., Brain B., Biga S., Schlebusch S., Dash P., Bowler S. D. (2013). Effect of long-term, low-dose erythromycin on pulmonary exacerbations among patients with non-cystic fibrosis bronchiectasis: the BLESS randomized controlled trial. *Journal of the American Medical Association*.

[27] Goeminne P. C., Soens J., Scheers H., De Wever W., Dupont L. (2012). Effect of macrolide on lung function and computed tomography (CT) score in non-cystic fibrosis bronchiectasis. *Acta Clinica Belgica*.

[28] Zheng L., Lam W. K., Tipoe G. L., Shum I. H., Yan C., Leung R., Sun J., Ooi G. C., Tsang K. W. (2002). Overexpression of matrix metalloproteinase-8 and -9 in bronchiectatic airways in vivo. *European Respiratory Journal*.

[29] Sepper R., Konttinen Y. T., Sorsa T., Koski H. (1994). Gelatinolytic and type IV collagenolytic activity in bronchiectasis. *Chest*.

[30] Martínez-García M. A., Soler-Cataluña J.-J., Perpiñá-Tordera M., Román-Sánchez P., Soriano J. (2007). Factors associated with lung function decline in adult patients with stable non-cystic fibrosis bronchiectasis. *Chest*.

[31] He Z.-Y., Ou L.-M., Zhang J.-Q., Bai J., Liu G.-N., Li M.-H., Deng J.-M., MacNee W., Zhong X.-N. (2010). Effect of 6 months of erythromycin treatment on inflammatory cells in induced sputum and exacerbations in chronic obstructive pulmonary disease. *Respiration*.

[32] Kanai K., Asano K., Hisamitsu T., Suzaki H. (2004). Suppresion in matrix metalloproteinase production from nasal fibroblasts by macrolide antibiotics in vitro. *European Respiratory Journal*.

[33] Zhong X.-N., Bai J., Shi H.-Z., Wu C., Liang G.-R., Feng Z.-B. (2003). An experimental study on airway inflammation and remodeling in a rat model of chronic bronchitis and emphysema. *Chinese Journal of Tuberculosis and Respiratory Diseases*.

